# Unusual Presentation of Diffuse Axonal Injury: A Case Report

**DOI:** 10.7759/cureus.31336

**Published:** 2022-11-10

**Authors:** Ahmed Alkhalifah, Mohammed Alkhalifa, Mohammed Alzoayed, Dunya Alfaraj, Rawan Makhdom

**Affiliations:** 1 Pediatric Medicine, Maternity and Children Hospital, Dammam, SAU; 2 Emergency Department, King Fahad University Hospital, Dammam, SAU

**Keywords:** dai, tbi, diffuse axonal injury, traumatic brain injury, head injury

## Abstract

Diffuse axonal injury (DAI) is the microscopic axonal injury in the brain’s neural pathways, corpus callosum, and brainstem, which has been linked to a major increase in morbidity and mortality. Patients present acutely with no lucid interval, abnormal pupil response, and decerebrate or decorticate posture. In this case report, we report on an 11-year-old girl who was agitated, crying, vomiting, had one episode of seizure, and had poor communication as a result of a high-risk mechanism side-impact motor vehicle collision (MVC).

## Introduction

Diffuse axonal injury (DAI) is the microscopic axonal injury in the brain’s neural pathways, corpus callosum, and brainstem, which has been linked to a major increase in morbidity and mortality. DAI is defined clinically by traumatic brain injury (TBI) causing coma lasting six hours or more, with the exception of cases of ischemic brain lesions or edema [1]. Patients present acutely with no lucid interval, abnormal pupil response, and decerebrate or decorticate posture [2]. The annual incidence rate of TBI globally is 200 per 100,000 people, leading to 40-50% of TBI patients developing DAI [3,4]. It is caused by acceleration-deceleration forces that cause axonal shearing and micro-hemorrhages in the grey-white junction in the cortex and white matter throughout the brain, which can be radiographically confirmed [5]. Tissue damage can be significant, with the involvement of several functional systems and multiple brain areas [6]. According to Gentry’s classification, DAI was classified into three stages [7]. Lesion involving the junction of the white and grey matter suggests stage I; corpus callosum involvement suggests stage II, and additional brainstem involvement suggests stage III [8]. An accurate and thorough radiologic evaluation would be beneficial in guiding treatment and predicting the final result [6]. Important advancements in neurologic imaging, particularly with brain magnetic resonance imaging (MRI), have been made, making DAI diagnosis easier [7]. Susceptibility-weighted imaging (SWI) is a high-resolution gradient-echo MRI technique and is highly useful in detecting hemorrhagic lesions associated with DAI [9]. In this study, we report on a patient who showed an unusual presentation of DAI with agitation, crying, and poor communication following a side-impact high-risk mechanism motor vehicle collision (MVC).

## Case presentation

**Figure 1:**
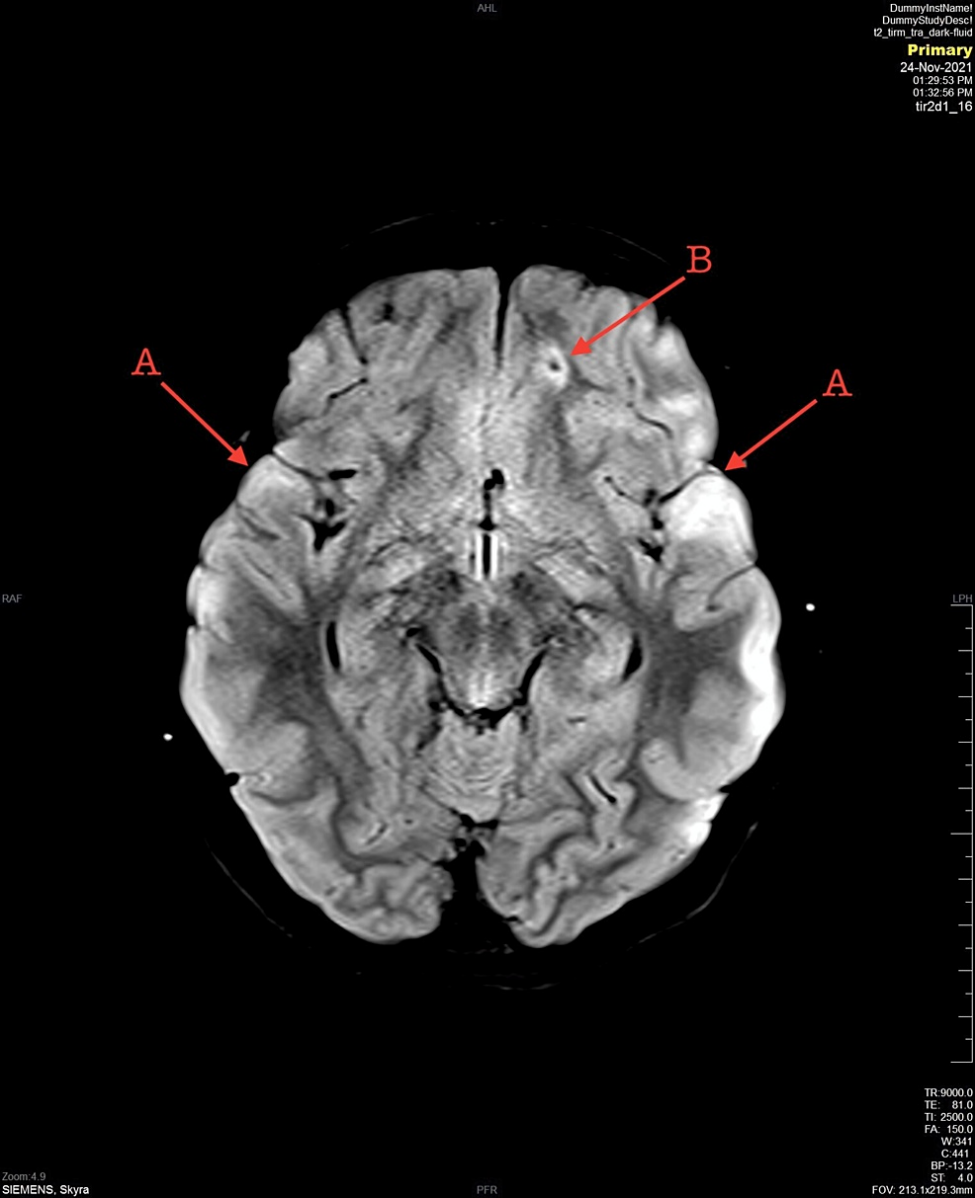
Bilateral hyperintense foci in the cortical (A) and subcortical (B) junction in fluid attenuated inversion recovery (FLAIR) MRI

**Figure 2:**
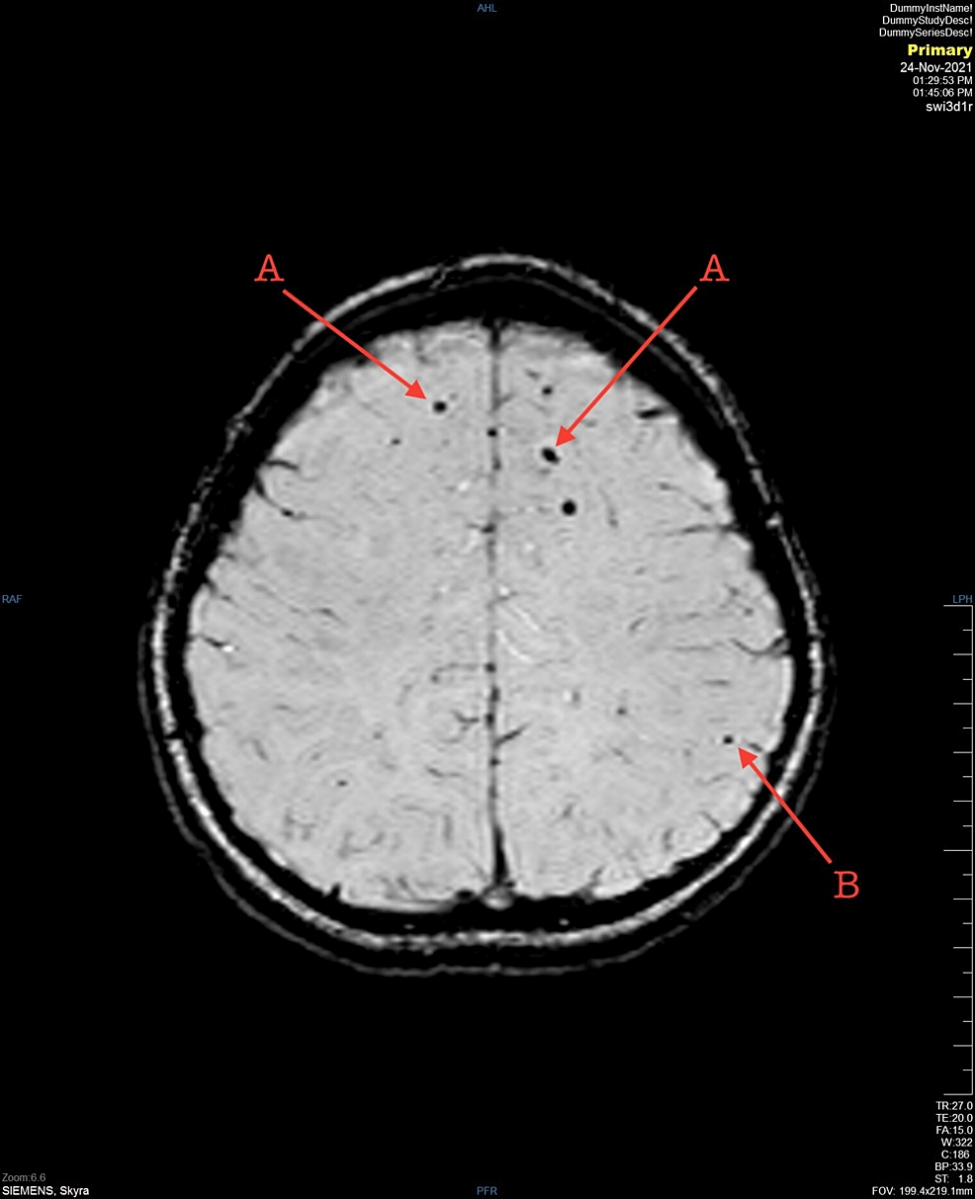
Bilateral frontal lobe (A) and left parietal lobe (B) with a hemorrhagic component in susceptibility-weighted imaging (SWI) sequence associated with multiple hyperintensities

**Figure 3:**
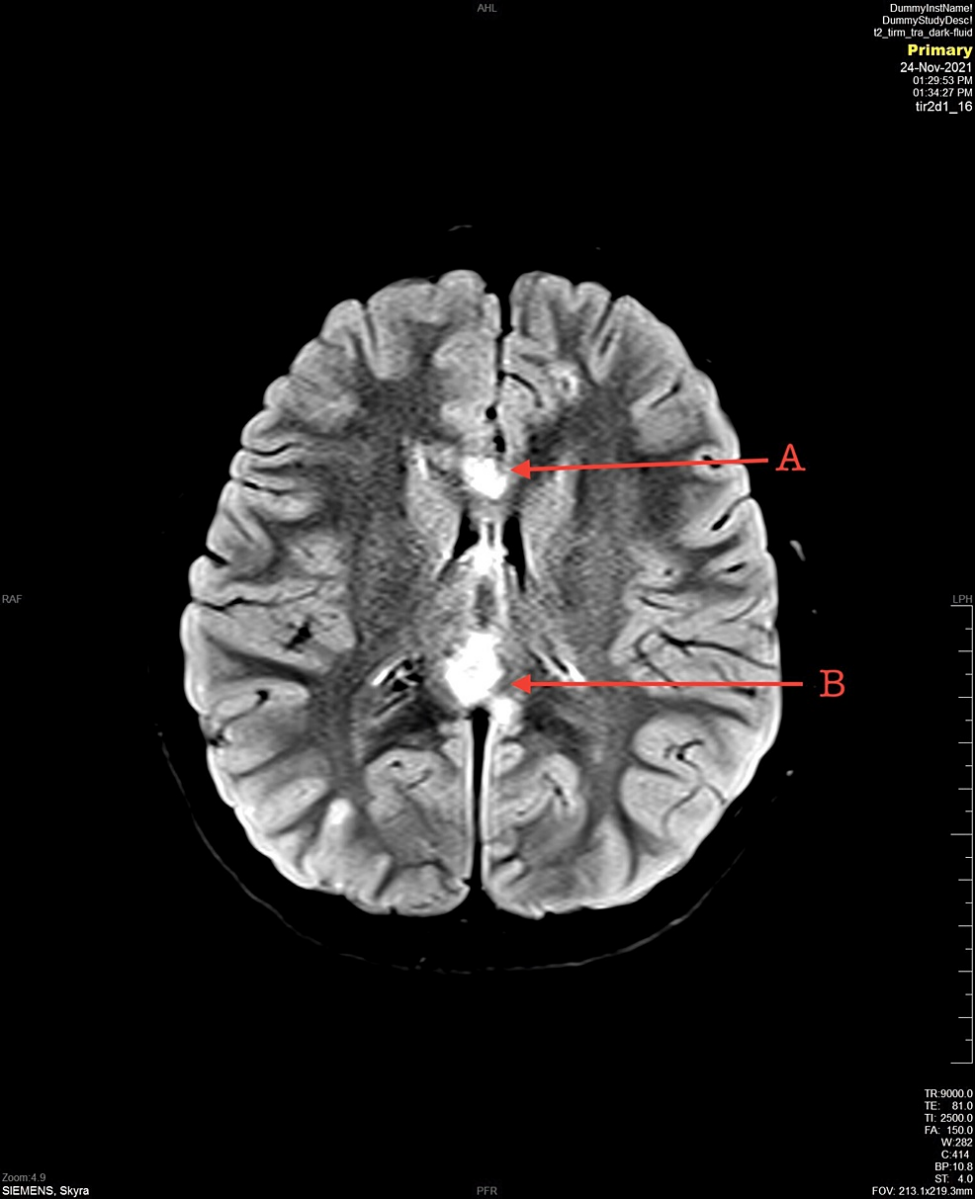
An abnormal fluid attenuated inversion recovery (FLAIR) signal intensity occupying the corpus callosum including genu (A) and splenium parts (B).

An 11-year-old girl with no medical issues was brought to the hospital by the Red Crescent as a result of an MVC. She was a passenger in a car hit in a high-risk mechanism side-impact MVC. Upon arrival at the Emergency Department, she was agitated, crying, vomiting, had one episode of seizure, and had poor communication as a result of the accident. A primary survey was done. The airway was patent, she was able to talk and handle secretion and a C-collar was applied. Auscultation was equal to bilateral air entry with oxygen saturation of 97%. Blood pressure was 116/80 and heart rate was 135. There were no signs of external bleeding, and stable pelvis and Extended Focused Assessment with Sonography in Trauma (E-FAST) was negative. Glasgow Coma Scale (GCS) was 15/15, the pupils were equally reactive, and she was able to move all limbs. On exposure, no midline tenderness or deformity was observed. The secondary survey was unremarkable. A non-enhanced computed tomography (CT) scan was done and showed suboptimal head CT. The patient received midazolam in the emergency room and was admitted under pediatric surgery care. On the third day of her hospital stay course, unenhanced multiplanar multi-sequential brain MRI was done and showed multiple bilateral hyper-intense foci seen in the cortical/subcortical junction (Figure 1) and both frontal lobe and left parietal lobe with a hemorrhagic component in SWI sequence associated with multiple hyperintensities noted (Figure 2). In addition, an abnormal fluid attenuated inversion recovery (FLAIR) signal intensity occupying the corpus callosum including genu and splenium parts likely represents grade 2 diffuse axonal injury (Figure 3). An electroencephalogram (EEG) was done on day three of admission and showed epileptiform discharges. A loading dose of phenytoin 20mg/kg over 30 minutes and levetiracetam 1300 mg every 12 hours (q12 H) were given, and the patient gradually recovered. Upon discharge, the patient was vitally and clinically stable without any evidence of any abnormal jerky movements. She was talking, tolerating orally, communicating, and walking freely. The discharge plan was to continue on phenytoin 130 mg twice daily (BID) and levetiracetam 1300 mg q12 H with an outpatient department follow-up after two weeks.

## Discussion

MVC may lead to a wide range of physical problems. The psychological state may be impacted by both psychogenic and medical effects such as cerebral trauma. The diagnosis of brain damage in vehicle accident victims presenting as DAI with rarely any physical effects appears to be a recent problem. Such harm gradually manifests as behavioral issues immediately following the event and significant memory loss later [10]. DAI clinical presentations at the emergency department can vary dramatically from case to case. For example, in our case the patient presented with agitation and crying, one episode of seizure, vomiting, and poor communication. In another case report, the patient presented with loss of consciousness and post-traumatic amnesia [11]. In addition, one case report showed that the patient was conscious and clinically stable with efficient respiration and circulation and a blunt injury to the left parietal region [10].

Patients with head trauma who are admitted to the emergency department require urgent evaluation by emergency doctors to rule out any trauma that needs a quick intervention to prevent rapid worsening of the patient’s health status [12]. Confabulation and attention deficits have a substantial negative influence on logical interaction with a patient and may deceive doctors, which can tip the diagnostic process in the direction of psychotic illnesses with delusions and disorganization [10].

Patients can arrive in a coma or lose consciousness. Clinical symptoms frequently do not reflect imaging results, and CT results could be unremarkable. Although high-speed car accidents are the most frequent cause of DAI, any type of head trauma, even very slight damage, can result in it [13]. Every imaging technique understates the severity of DAI. CT scans cannot detect diffuse brain damage. Only 20% to 50% of patients with axonal shear injuries display abnormal findings on CT scans of acute head traumas, partly because of their small size and common initial non-hemorrhagic character [13].

When it comes to identifying shear damage, MRI is more sensitive than other methods. Imaging should be done on individuals with suspected DAI at least three to seven days after the injury since this is when necrosis and edema in the nerves are most common [13]. Emergency physicians must keep high clinical suspicion of DAI among head trauma victims who present with poor communication and abnormal behavior. If CT head is normal for definitive diagnosis and proper management, requesting an MRI is encouraged.

## Conclusions

Diffuse axonal injury is a serious primary traumatic brain injury following acceleration-deceleration forces. DAI should be suspected when behavioral problems and memory deficits with features of disorganization and disinhibition are present without considerable focal symptoms. Clinical presentations of DAI can vary dramatically from case to case. SWI technique and FLAIR sequence are highly useful in detecting lesions associated with DAI.
